# Partial Deficiency of Zfp217 Resists High-Fat Diet-Induced Obesity by Increasing Energy Metabolism in Mice

**DOI:** 10.3390/ijms22105390

**Published:** 2021-05-20

**Authors:** Qianhui Zeng, Nannan Wang, Yaru Zhang, Yuxuan Yang, Shuangshuang Li, Rong Zheng, Jin Chai, Tong Qiao, Siwen Jiang

**Affiliations:** Key Laboratory of Animal Genetics, Breeding and Reproduction Ministry of Education, College of Animal Science and Technology, Huazhong Agricultural University, Wuhan 430070, China; zengqianhui2021@163.com (Q.Z.); genewangnannan@gmail.com (N.W.); z15563102012@163.com (Y.Z.); yangyuxuan261@163.com (Y.Y.); lishuangshuang188@163.com (S.L.); zhengrong@mail.hzau.edu.cn (R.Z.); chaijin@mail.hzau.edu.cn (J.C.); qiaotong2021@163.com (T.Q.)

**Keywords:** Zfp217, obesity, adipogenesis, glucose tolerance, insulin sensitivity, energy expenditure

## Abstract

Obesity-induced adipose tissue dysfunction and disorders of glycolipid metabolism have become a worldwide research priority. Zfp217 plays a crucial role in adipogenesis of 3T3-L1 preadipocytes, but about its functions in animal models are not yet clear. To explore the role of Zfp217 in high-fat diet (HFD)-induced obese mice, global Zfp217 heterozygous knockout (Zfp217^+/−^) mice were constructed. Zfp217^+/−^ mice and Zfp217^+/+^ mice fed a normal chow diet (NC) did not differ significantly in weight gain, percent body fat mass, glucose tolerance, or insulin sensitivity. When challenged with HFD, Zfp217^+/−^ mice had less weight gain than Zfp217^+/+^ mice. Histological observations revealed that Zfp217^+/−^ mice fed a high-fat diet had much smaller white adipocytes in inguinal white adipose tissue (iWAT). Zfp217^+/−^ mice had improved metabolic profiles, including improved glucose tolerance, enhanced insulin sensitivity, and increased energy expenditure compared to the Zfp217^+/+^ mice under HFD. We found that adipogenesis-related genes were increased and metabolic thermogenesis-related genes were decreased in the iWAT of HFD-fed Zfp217^+/+^ mice compared to Zfp217^+/−^ mice. In addition, adipogenesis was markedly reduced in mouse embryonic fibroblasts (MEFs) from Zfp217-deleted mice. Together, these data indicate that Zfp217 is a regulator of energy metabolism and it is likely to provide novel insight into treatment for obesity.

## 1. Introduction

Obesity is a key risk factor for many chronic diseases, including type 2 diabetes, cardiovascular disease, and cancer [1,2,3,4]. When energy intake exceeds energy expenditure, the body stores energy mainly as triglycerides, which leads to excessive growth of adipose tissue [5]. Principal characteristics of obesity are adipocyte hypertrophy and hyperplasia [6]. With the worldwide obesity epidemic, it remains important to study adipocyte growth and development, adipose tissue regulation of energy homeostasis, and glucose homeostasis.

Adipose tissue is an important metabolic organ, whose main function is an energy storage site under conditions of energy excess [7] and can also secrete several endocrine hormones such as adiponectin [7,8]. Adipose tissue mediates the regulation of glucose lipid homeostasis and is essential for systemic insulin sensitivity and energy balance [9,10]. Adipose tissue consists mainly of adipocytes, which undergo a multi-step directed process to generate pre-adipocytes when they encounter stimuli, which are subsequently activated by many regulatory factors (signaling pathways and transcription factors) under lipogenesis-inducing conditions to differentiate into adipocytes [11,12,13]. Several reviews have systematically summarized the molecular regulatory mechanisms of adipogenesis and constructed a transcriptional cascade regulatory network [14,15,16]. There are two major adipogenic factors at the core of this network, PPARγ and C/EBPα, which play a key role in systemic lipid and glucose metabolism [17,18,19].

Numerous studies have shown that zinc finger protein (ZFP) has an essential role in fat biology [20]. For example, KLF5 knockout mice are protected against high-fat diet-induced obesity, with impaired adipogenesis in mouse embryonic fibroblasts (MEFs) of KLF5^+/−^ mice [21,22]. Zfp407 regulates glucose homeostasis and insulin sensitivity by regulating GLUT4 transcript levels and PPARγ activity in response to insulin stimulation [23]. Zinc Finger Protein 217 (Zfp217) belongs to the Krüppel-type transcription factors [24]. Recent studies have revealed that Zfp217 is also involved in adipogenesis, through interaction with histone methylation enzyme EZH2 to promote adipogenesis by influencing cell cycle [25]; through the interaction of Zfp217 and m6A methyltransferase METTL3 or demethylase FTO, the m6A modification level of related downstream target genes is influenced and mediates post-transcriptional modifications of m6A in a YTHDF2-dependent manner, thereby affecting adipocyte differentiation, suggesting that Zfp217 could play an important role in adipogenesis at the level of transcription and post-transcriptional modifications [26,27]. However, the function of Zfp217 in in vivo animal models is largely unknown.

In the present study, using a novel heterozygous mouse model, we observed that partial deficiency of Zfp217 mice fed high-fat diets presented less body weight accompanied by improved glucose tolerance, increased insulin sensitivity, and energy expenditure. Accordingly, these data show that Zfp217 functions as a regulator of systemic energy metabolism in a live animal model.

## 2. Results

### 2.1. Generation of Zfp217 Heterozygote Mice

**Figure 1:**
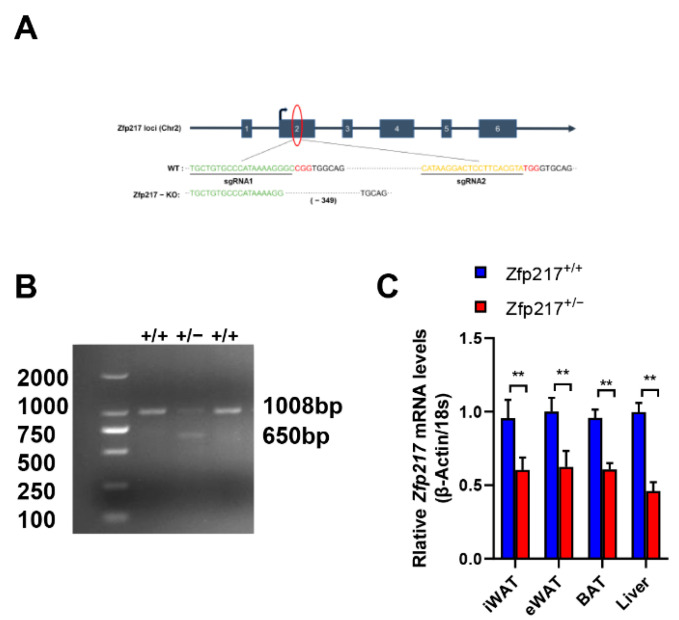
Generation of whole-body Zfp217 knockout mice. (**A**) Schematic of a gene-targeting strategy to create Zfp217 KO mice. (**B**) PCR-based genotyping of Zfp217 KO and WT alleles. (**C**) Quantitative real-time PCR analysis of Zfp217 mRNA expression in inguinal white adipose tissue (iWAT), epididymal white adipose tissue (eWAT), brown adipose tissue (BAT), and liver of Zfp217^+/+^ and Zfp217^+/−^ mice (*n* = 8 per group). For all statistical plots, data are presented as the mean ± SEM. ** *p* < 0.01.

To elucidate the physiological role of Zfp217 in vivo, whole-body Zfp217-knockout mice were generated using the CRISPR/Cas9 system (Figure 1A). We also performed a test for the probability of sgRNA off-target before the start of the experiment. Results from agarose gel electrophoresis showed no genome editing in any of the five most probable off-target sites (Figure S4). To generate homozygous Zfp217 knockout mice, we intercrossed heterozygous Zfp217^+/−^ mice in the earlier experiments. Unfortunately, we found Zfp217 homozygous null mice are embryonically lethal and no live mice were detected. According to the previous literature, we learned that knockout Zfp217 has effects on exiting from pluripotency in embryonic stem cell (ESC) differentiation, possibly because Zfp217 expression is rapidly decreased and METTL3 is released to maintain of m^6^A methylation of pluripotent transcripts, which causes embryonic stem cell differentiation [28], or NuRD mediates H3K27 acetylation and PCR2 mediates H3K27 trimethylation to silence the ESC differentiation-related gene [29]. The causes of lethality in Zfp217 homozygous null embryos, however, remain unclear. To circumvent this problem, we used Zfp217^+/−^ mice in the present study. Inter-crossing of heterozygote mice with WT (wild type) mice yielded Zfp217 knockout (Zfp217^+/−^) and WT (Zfp217^+/+^) littermates. Genotypes of mice were identified through PCR (Figure 1B). The distribution of genotypes was in accordance with the Mendelian ratio, and the appearance of the newborns was normal. Subsequently, quantitative real-time PCR analysis on inguinal white adipose tissue (iWAT), epididymal white adipose tissue (eWAT), brown adipose tissue (BAT), and liver showed that Zfp217 mRNA levels were approximately 50% lower in the Zfp217^+/−^ mice compared to Zfp217^+/+^ littermates (Figure 1C). These results showed that the model of Zfp217 systemic knockout mice was generated successfully.

### 2.2. Zfp217^+/−^ Mice Exhibit a Similar Phenotype to Zfp217^+/+^ Mice under a Normal Chow Diet (NC)

**Figure 2:**
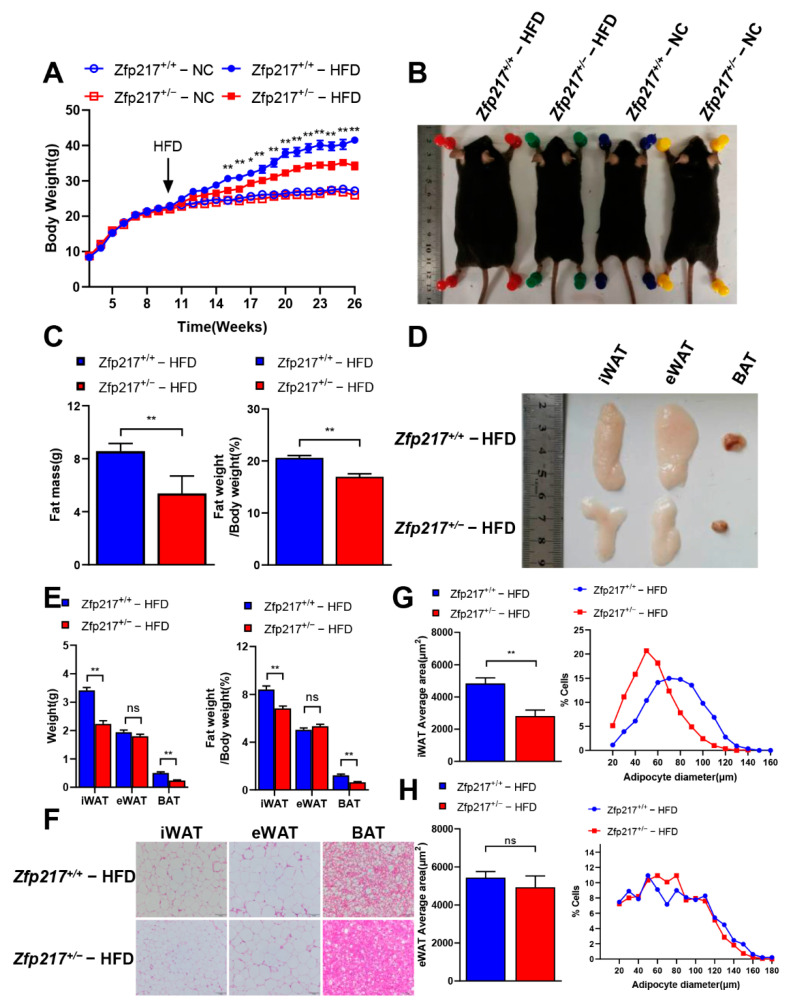
Zfp217^+/−^ mice are resistant to high-fat diet-induced obesity. (**A**) Body weight of Zfp217^+/+^ and Zfp217^+/−^ mice fed a normal chow diet (NC) for 10 weeks and then switched to a high-fat diet (HFD) for 16 weeks (*n* = 8 per group). (**B**) External appearance of representative Zfp217^+/+^ and Zfp217^+/−^ mice shown in A at 26 weeks. (**C**) Body fat weight or percentage of Zfp217^+/+^ and Zfp217^+/−^ mice (*n* = 8 per group). (**D**) Gross appearance of adipose tissues from representative Zfp217^+/+^ and Zfp217^+/−^ mice shown in A at 26 weeks. (**E**) iWAT, eWAT, BAT weight or percentage of Zfp217^+/+^ and Zfp217^+/−^ mice (*n* = 8 per group). (**F**) H&E staining of adipose tissue from Zfp217+/+ and Zfp217^+/−^ mice (Scale bar: 100 μm) (*n* = 6 per group). (**G**,**H**) Average cell area (**left**) and adipocyte size distribution (**right**) in iWAT (**G**) and eWAT (**H**) of Zfp217^+/+^ and Zfp217^+/−^ mice (*n* = 6 per group). For all statistical plots, data are presented as the mean ± SEM. * *p* < 0.05; ** *p* < 0.01; ns indicates no significance between the two indicated groups.

When maintained under a normal chow diet (NC) ad libitum, the Zfp217^+/−^ mice had similar body weight and food intake to Zfp217^+/+^ mice (Figure 2A; Supplemental Figure S1A). Moreover, the body composition of the NC-fed Zfp217^+/−^ mice had a similar gross appearance and percentage total body fat as Zfp217^+/+^ mice (Figure 2B; Supplemental Figure S1B). iWAT, eWAT, and BAT pads were not significant between the Zfp217^+/+^ mice and Zfp217^+/−^ mice (Supplemental Figure S1C,D). Histological observations also confirmed no obvious differences in sections of various adipose tissues (Supplemental Figure S1E–G) of the Zfp217^+/+^ mice and Zfp217^+/−^ mice, regardless of the average area of adipocytes or the distribution of adipocyte diameters.

Furthermore, glucose homeostasis in the mice was assessed by glucose and insulin tolerance tests. The GTTs and ITTs revealed no differences between the Zfp217^+/+^ mice and Zfp217^+/−^ mice (Supplemental Figure S2A–D). In addition, we also measured the oxygen consumption (VO2) and CO2 production (VCO2) rates of both genotypes by indirect calorimetry to determine energy expenditure. The physical activity and respiratory exchange ratio were comparable between genotypes (Supplemental Figure S3A,B). Consistent with the physical activity, Zfp217^+/−^ mice showed similar VO2 and VCO2 as well as energy expenditure in light phases when compared with Zfp217^+/+^ mice (Supplemental Figure S3C,D), but Zfp217^+/−^ mice showed increased VO2 as well as increased energy expenditure in dark phases when compared with Zfp217^+/+^ mice (Supplemental Figure S3C–E). Collectively, these data provide evidence that Zfp217^+/−^ mice had no defective adipocyte differentiation ability and normal adipose tissues under NC. Possibly because we used a constitutive whole-body heterozygous knockout mice model, the possibility that compensatory responses mask the requirement for Zfp217 in metabolic homeostasis in the basal state cannot be excluded. Future studies employing conditional KO of Zfp217 in adult mice will help address this issue.

### 2.3. Zfp217 Knockout Mice Are Resistant to Diet-Induced Obesity

To investigate the effects of high-fat diet (HFD) treatment, a cohort of 10-week-old Zfp217^+/+^ and Zfp217^+/−^ male mice were fed ad libitum a diet containing 60% fat for 16 weeks. Interestingly, the Zfp217^+/−^ mice displayed prevented HFD-induced obesity, and their body-weight gain was significantly slower than that of the Zfp217^+/+^ mice after 16 weeks of HFD feeding (Figure 2A). However, there was no difference in food intake between Zfp217^+/+^ mice and Zfp217^+/−^ mice (Supplemental Figure S1A). We found that Zfp217^+/−^ mice had a smaller gross appearance than that of Zfp217^+/+^ mice (Figure 2B). We found that HFD-fed Zfp217^+/−^ mice had an extremely significantly lower fat weight and percentage total body fat than those of Zfp217^+/+^ mice (Figure 2C). Further analysis revealed that iWAT and BAT pads were smaller in Zfp217^+/−^ mice than in Zfp217^+/+^ mice, but eWAT pads showed no difference between Zfp217^+/+^ mice and Zfp217^+/−^ mice (Figure 2E). Subsequently, sections of iWAT and BAT of Zfp217^+/+^ mice and Zfp217^+/−^ mice showed obvious differences by histological observation, but not eWAT (Figure 2F). To determine whether the reduction in the size of the fat pad was because of fewer cell numbers or smaller adipocytes, we examined the inguinal fat tissue sections and found that the average area and the size distribution of adipocyte were smaller in the Zfp217^+/−^ mice adipose tissues than those of the Zfp217^+/+^ mice (Figure 2G). However, we found that the average area of adipocytes and the distribution of adipocyte diameters did not differ in the eWAT of Zfp217^+/+^ mice and Zfp217^+/−^ mice (Figure 2H). A comparison between the average area of inguinal adipocytes (Figure 2H) and the average inguinal fat pad weight (Figure 2E) suggested that the reduced total fat accumulation in Zfp217^+/−^ mice was primarily because of the reduced size of the inguinal adipocytes.

**Figure 3:**
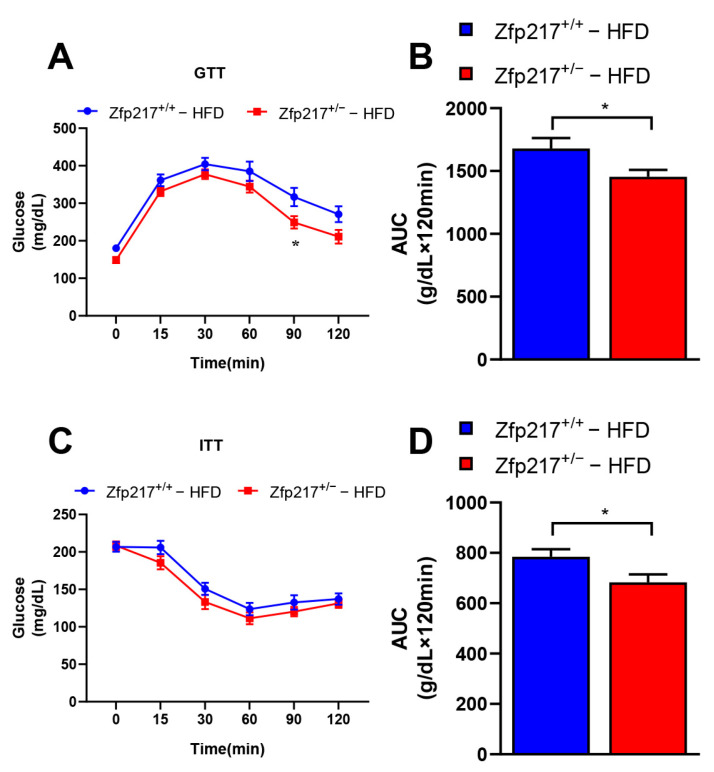
Zfp217 deficiency improves glucose tolerance and insulin sensitivity under HFD. (**A**,**B**) IP glucose tolerance tests (GTTs) were performed on Zfp217^+/+^ and Zfp217^+/−^ mice at 14 weeks, and the corresponding areas under the curves were calculated (*n* = 8 per group). Comparison at each time point was made against Zfp217^+/+^ control mice by two-way ANOVA. (**C**,**D**) IP insulin tolerance test (ITT) was performed on Zfp217^+/+^ and Zfp217^+/−^ mice at 15 weeks, and the corresponding areas under the curves were calculated (*n* = 8 per group). Comparison at each time point was made against Zfp217^+/+^ control mice by two-way ANOVA. For all statistical plots, data are presented as the mean ± SEM. * *p* < 0.05.

A high-fat diet causes disturbances in glucose metabolism in mice [30]. The GTT and ITT reflect the body’s ability to regulate glucose and the peripheral tissue’s sensitivities response to insulin, respectively [31,32]. The GTT results showed that the Zfp217^+/−^ mice exhibited improved glucose clearance compared with Zfp217^+/+^ mice (Figure 3A,B). Correspondingly, Zfp217^+/−^ mice were also more sensitive to insulin than Zfp217^+/+^ mice, as determined by ITT (Figure 3C,D). Together, these data suggest that loss of Zfp217 expression ameliorates HFD-induced obesity-related metabolic syndrome including insulin resistance and glucose intolerance.

### 2.4. Zfp217 Knockout Mice Demonstrate Increased Energy Expenditure

**Figure 4:**
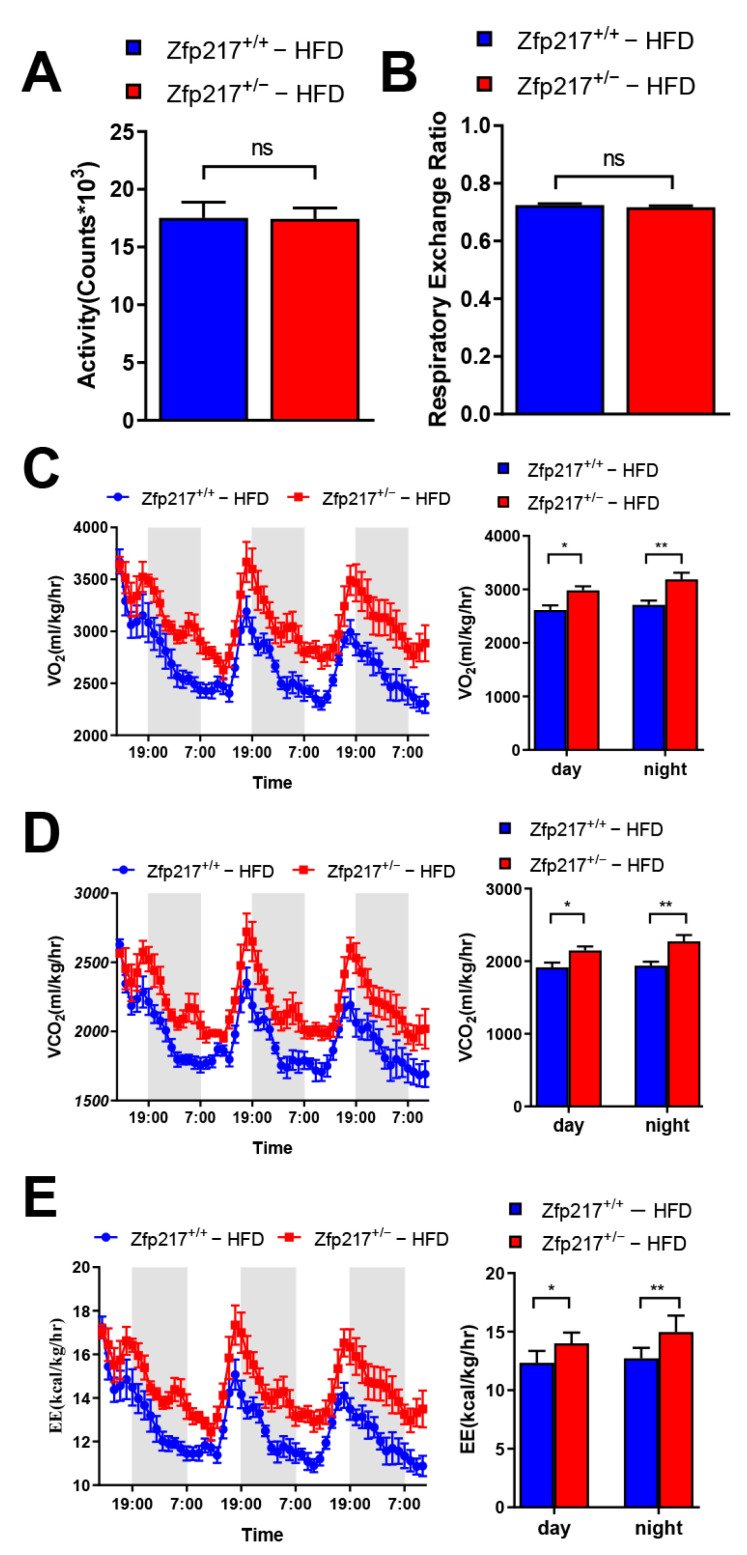
Zfp217 deficiency increases energy expenditure under HFD. (**A**) Physical activity, (**B**) Respiratory exchange ratio (**C**) Oxygen consumption (VO_2_), (**D**) Carbon dioxide generation (VCO_2_), and (**E**) Energy consumption analyzed by indirect calorimetry in Zfp217^+/+^ and Zfp217^+/−^ mice after HFD feeding at 25 weeks (*n* = 6 per group). For all statistical plots, data are presented as the mean ± SEM. * *p* < 0.05; ** *p* < 0.01; ns indicates no significance between the two indicated groups.

To investigate whether Zfp217 deficiency might cause alterations in energy metabolism, we next measured the mice for several metabolic parameters. Surprisingly, indirect calorimetry analysis revealed that the total volume of carbon dioxide production (VCO_2_) and oxygen consumption (VO_2_) were significantly higher in HFD-fed Zfp217^+/−^ mice than in Zfp217^+/+^ mice during both light and dark phases (Figure 4C,D). Correspondingly, energy expenditure rate (EE) was significantly increased in Zfp217^+/−^ mice during both light and dark compared to Zfp217^+/+^ mice (Figure 4E). Since no differences were observed in physical activity or the respiratory exchange ratio (Figure 4A,B), we therefore concluded that higher energy expenditure in HFD-fed Zfp217^+/−^ mice is the primary mechanism facilitating their resistance to adiposity and body-weight gain.

### 2.5. Impact of Zfp217 Deletion on Gene Expression in Inguinal Adipose Tissue

**Figure 5:**
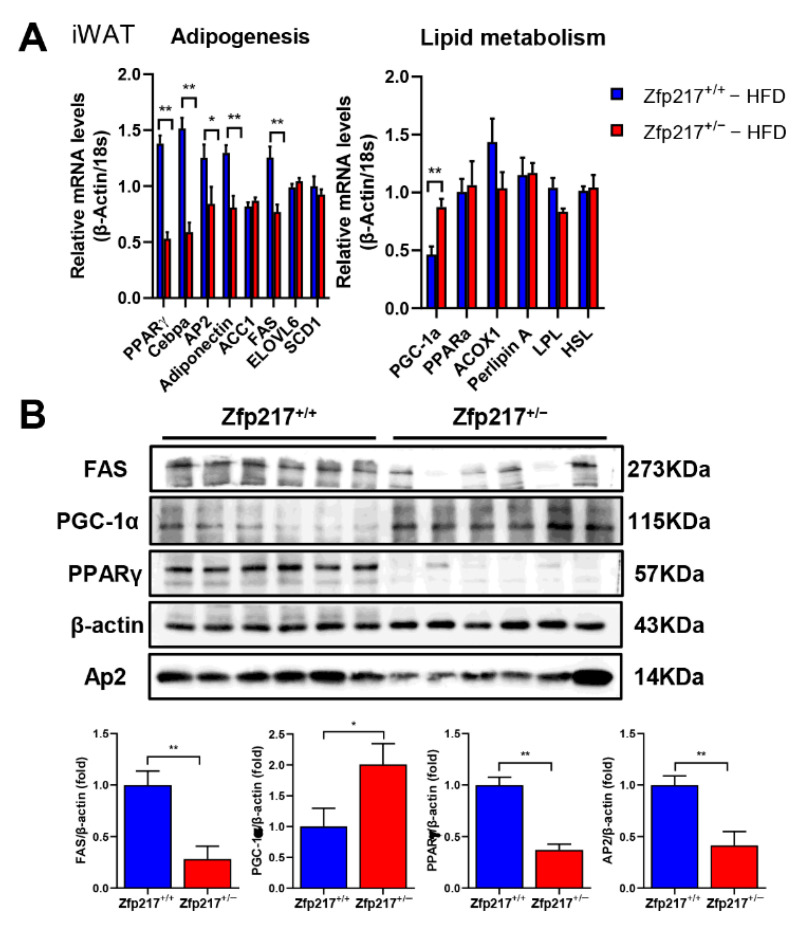
Gene expression in the iWAT of Zfp217^+/+^ and Zfp217^+/−^ mice fed a high-fat diet. (**A**) Quantitative real-time PCR analysis of genes involved in adipogenesis and lipid metabolism in iWAT of Zfp217^+/+^ and Zfp217^+/−^ mice fed an HFD. Expression data were normalized to β-actin and the 18 S geometric mean in each sample (*n* = 6 per group). (**B**) Western blotting analysis of genes involved in adipogenesis and lipid metabolism in iWAT of Zfp217^+/+^ and Zfp217^+/−^ mice fed an HFD. Expression data were normalized to the β-actin geometric mean in each sample (*n* = 6 per group). For all statistical plots, data are presented as the mean ± SEM. * *p* < 0.05; ** *p* < 0.01.

Our previous study showed that Zfp217 is positively correlated with triglyceride accumulation and can promote fat accumulation through post-transcriptional modification of m6A [25,27]. Similarly, we found that Zfp217 deletion causes resistance to HFD-induced obesity in in vivo experiments, possibly due to reduced adipogenesis or increased metabolic thermogenesis. To test the possibility, we selected the most important genes related to adipogenesis and lipid metabolism. Subsequently, we examined their expression in the inguinal fat tissue of Zfp217^+/+^ and Zfp217^+/−^ mice fed an HFD for 16 weeks. We found that the mRNA expression levels of adipogenesis-related genes (PPARγ, CEBPα, AP2, adiponectin, and FAS) in the inguinal adipose tissue of high-fat-fed Zfp217^+/−^ mice were significantly lower than those of Zfp217^+/+^ mice, whereas the mRNA expression levels of lipid metabolism-related genes (PGC-1α) were extremely significantly higher than those of Zfp217^+/+^ mice (Figure 5A). At the same time, we also found that the protein expression of adipogenesis-related genes (PPARγ, AP2, and FAS) and lipid metabolism-related genes (PGC-1α) showed the same trend (Figure 5B). The data suggest that the reduction of Zfp217 activity can alter the expression of genes and inhibit impairment of adipose tissue lipid storage. The differential expression of genes may be the molecular cause of resistance to high-fat diet-induced obesity.

### 2.6. Zfp217 Knockout Mice Exhibit Reduced Adipogenesis

**Figure 6:**
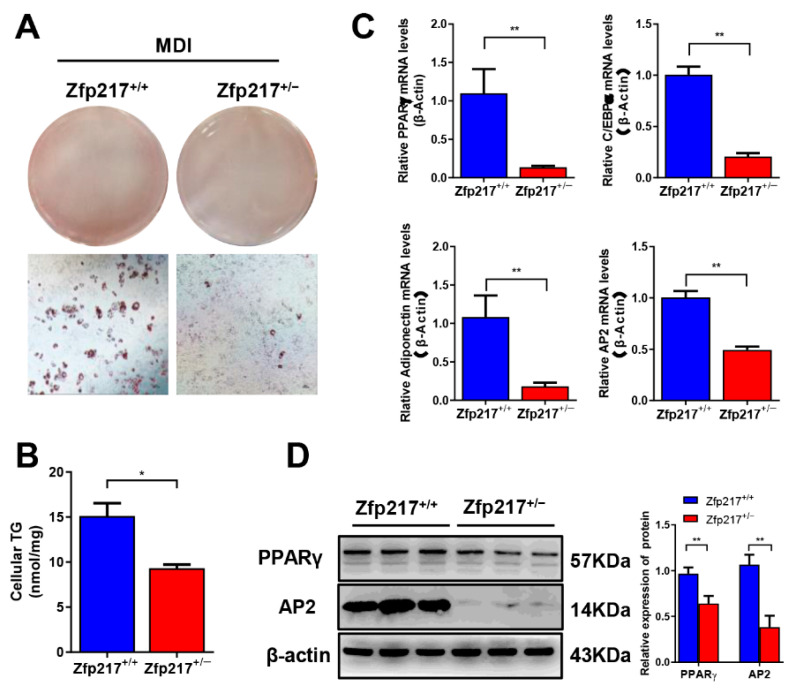
Zfp217 deficiency impaired adipogenesis in vitro. (**A**) Oil red-O staining of lipids in differentiated cells (Scale bar: 100 μm) (*n* = 3 per group). (**B**) A triglyceride assay kit was used for analysis of the content of triglycerides (*n* = 3 per group). (**C**) mRNA expression levels of PPARγ, C/EBPα, adiponectin, and AP2 in differentiated cells were measured by quantitative real-time PCR analysis (*n* = 3 per group). (**D**) Protein expression of Zfp217, PPARγ, and AP2 in differentiated cells were measured by Western blotting (*n* = 3 per group). For all statistical plots, data are presented as the mean ± SEM. * *p* < 0.05; ** *p* < 0.01.

To determine the role of Zfp217 in adipogenesis in Zfp217 knockout mice, we examined adipogenic differentiation potential in vitro. MEF cells were isolated from an E14.5 d embryo and induced in an adipogenic culture medium as described in Materials and Methods. The differentiation was assessed by examining lipid accumulation and determining gene expression. Compared to Zfp217^+/+^ cells, Oil Red O staining demonstrated that Zfp217^+/−^ MEF had fewer lipid droplets (Figure 6A). This result was similar to that of the triglyceride content measurement. The triglyceride content in differentiated adipocytes from Zfp217^+/−^ MEF was significantly lower than that in Zfp217^+/+^ cells (Figure 6B). These results suggest a decreased adipogenic potential in Zfp217-deficient cells. We examined the expression of transcription factors (PPARγ and C/EBPα) that are required for adipocyte differentiation. After differentiation, Zfp217^+/−^ cells had an extremely significant reduction in mRNA and protein for PPARγ and AP2 (Figure 6C,D). There were also extremely significant changes in mRNA for C/EBPα and adiponectin (Figure 6C). The results imply that Zfp217 deletion inhibits adipogenesis of MEFs cells, which is consistent with the in vivo level data.

## 3. Discussion

A series of factors have been reported to be involved in the process of adipocyte differentiation, and the molecular regulatory mechanisms of adipogenesis have been systematically summarized [14,33]. PPARγ and C/EBPα have emerged as master regulators of adipogenesis, which oversee the entire terminal differentiation process [34,35]. Understanding the transcriptional cascade regulatory network of these factors and the mechanisms regulating adipogenesis is of great significance for the treatment of obesity.

It is well known that ZFP217, which a member of the histone complex repressors, is involved in many biological processes, including tumorigenesis and embryonic development [28,36,37]. Recently, it has been reported that Zfp217 levels are positively associated with triglycerides, and knockdown of Zfp217 hinders adipogenic differentiation by reducing the expression of key lipogenic genes PPARγ, C/EBPα, Ap2, and adiponectin in 3T3-L1 cells, which means Zfp217 has a potential effect on obesity [25,26]. Based on these results, to better understand the impact of Zfp217 loss on obesity, we sought to address the effect of Zfp217 deficiency on metabolic homeostasis by establishing a novel mouse model heterozygous for the Zfp217 gene using the CRISPR/Cas9 system. In our study, we found that the detection of adipogenesis-related genes PPARγ, C/EBPα, Ap2, and adiponectin in iWAT and MEFs showed a significant decrease after Zfp217 deletion (Figure 5A and Figure 6C,D). This is consistent with previous research. These data imply that the expression of Zfp217 is significantly positively correlated with the expression of adipogenic genes such as PPARγ, C/EBPα, and Ap2. In vitro studies and mice models with high fat diets showed that Zfp217 plays a key role in adipogenesis.

Consistent excessive caloric intake usually leads to increased fat deposition, mainly in the subcutaneous fat cells [38]. In addition, there are also reports in the literature that white adipose tissue can be divided into subcutaneous adipose depots (e.g., inguinal adipose tissue) and visceral adipose tissue (e.g., epididymal adipose tissue) according to anatomical location [39]. In obesity, visceral fat can expand by increasing the size of adipocytes, while subcutaneous fat enlarges mainly by increasing the size or number of adipocytes to adapt and expand to excess energy [40,41]. Simultaneously, our study further confirms that Zfp217^+/−^ mice can resist obesity because they have smaller and fewer adipocytes in inguinal adipose tissue in vivo (Figure 2A,D–G). Our results are in agreement with those reported earlier. Once adiposity accumulates, adipose tissue dysfunction leads to the production of a series of metabolic syndromes, including insulin resistance as the dominant feature [42,43]. In this study, we found that obese Zfp217^+/+^ mice had significantly poorer glucose tolerance and insulin sensitivity than Zfp217^+/−^ mice (Figure 3). Our data further indicate that Zfp217 knockout mice had reduced body fat, which protected against high-fat diet-induced insulin resistance.

In general, changes in body weight are closely related to physical activity and energy-producing metabolism besides the body’s feeding activity and material metabolism [30]. In the present study, we found that obesity in Zfp217^+/+^ mice was not because of their excessive energy intake or less physical activity than that of Zfp217^+/−^ mice (Figure 4A and Figure S1A). It has been reported that total oxygen consumption is reduced and mitochondrial activity is decreased under obese condition [44]. As observed in this experiment, Zfp217^+/+^ mice fed high-fat diets showed a significant decrease in oxygen consumption, but Zfp217^+/−^ mice could recover this phenotype (Figure 4C). Surprisingly, we newly found that Zfp217 can affect the expression of PGC-1α, which resists obesity by increasing the thermogenic capacity of the body. (Figure 5A,B), in addition to participating in the adipogenic process. It is now well established that PGC-1α acts as a transcriptional cofactor involved in a variety of biological processes including adaptive thermogenesis, mitochondrial formation, glucose and fatty acid metabolism and is closely associated with diseases such as obesity and type 2 diabetes [45,46,47]. Previously reported functions of Zfp217 were mainly related to lipogenic differentiation, and this provides new ideas and insights into our understanding of Zfp217 regulation of obesity.

## 4. Conclusions

In conclusion, we describe the effects of Zfp217 deficiency on whole-body glucose and insulin levels and thermogenesis in a basal state and in diet-induced obesity. We found that Zfp217^+/−^ mice were comparable to Zfp217^+/+^ mice on a normal chow diet, but Zfp217^+/−^ mice were resistant to high-fat diet-induced obesity and were insulin resistant with improved glucose hemostasis. The present study showed that Zfp217 could function by regulating related genes acting on both adipogenesis and lipid metabolism pathways, but the specific molecular mechanisms still need to be investigated in depth. Therefore, our results highlight that Zfp217 may be a novel target to reduce obesity and its related complications in patients.

## 5. Materials and Methods

### 5.1. Generation of Genetically Modified Mice

Whole-body Zfp217-knockout C57BL/6J mice were constructed via CRISPR/Cas9 technology. sgRNAs targeting the mouse Zfp217 were designed using the CRISPR on-line design tool (http://crispr.mit.edu/, accessed on 22 April 2021). sgRNAs sequences were as follows: sgRNA1: TGCTGTGCCCATAAAAGGGC, sgRNA2: CATAAGGACTCCTTCACGTA. Off-target analysis for sgRNA2 and amplifications for each primer pair (Table S2) were carried out separately. The PCR products were incubated at 37 °C for 15 min with T7E1 enzyme and were examined by 1.5% agarose gel. The sgRNAs and Cas9 nuclease were injected into fertilized oocytes. In this study, we generated 12 heterozygous knockout mice by CRISPR/Cas9 technology. Six of these mice (#1, #4, #6, #7, #12, and #14) had deletions of >300 bp fragment, while the remaining six mice (#2, #8, #9, #10, #11, and #13) only had <50 bp fragment deletions. For easy genotype identification and maximum speed of breeding, we selected male mice with >300 bp deletion (#12) as the F0 generation germline mice. The heterozygous KO (Zfp217^+/^^−^) and wild-type (Zfp217^+/+^) littermates were used in the study. These mice were maintained at 23 ± 1 °C with a 12 h light/dark cycle and were housed in plastic boxes with free access to water and diet. All procedures were performed in accordance with the institutional animal care and use committees at Huazhong Agricultural University.

### 5.2. Diets

An obesity murine model was established by feeding a normal chow diet (NC; 10% fat, 70% carbohydrates, and 20% protein; D12450B) for 10 weeks and then switched to a high-fat diet (HFD; 20% protein, 60% fat, and 20% carbohydrates; D12492) for 16 weeks. Mice that were fed an NC post weaning until 26 weeks of age served as controls. The feed intake and body weight were recorded weekly.

### 5.3. Genotype Identification

Mice were weaned at three weeks of age. DNA was prepared from tail samples using the Mini BEST Universal Genomic DNA Extraction Kit Ver 5.0 according to the manufacturer’s instructions (Japan, TAKARA, #9765). PCR analysis was used for genotyping and was performed with a Bio-Rad machine. Zfp217-KO and wild-type alleles were detected by PCR assays in which primer F1 (5′-TCGTGCTGACGCACATCTGACTC-3′) and primer R1 (5′-GGGTTCCTCTCGGTGGTCATCAG-3′) amplified a 1008-bp fragment (WT) and a 650-bp fragment (Zfp217-KO).

### 5.4. Tissue Isolation

At 26 weeks of age, the mice were fasted for 12 h and sacrificed under anesthesia. Samples included inguinal fat, epididymal fat, brown fat, and liver. Samples were fixed in 4% paraformaldehyde at room temperature and processed for histology. The frozen samples for protein and mRNA analysis were kept at −80 °C.

### 5.5. Glucose and Insulin Tolerance Test

Intraperitoneal glucose tolerance test (IP-GTT) and intraperitoneal insulin tolerance test (IP-ITT) were performed as previously described [48]. The process as follows: IP-GTT and IP-ITT were performed after 14 and 15 weeks of NCD or HFD feeding, respectively. The fasting blood glucose levels (t = 0) were measured using a glucometer (Roche, ACCU-Chek active) after either 16 h for IP-GTT or 6 h for IP-ITT. Then, glucose (2 g/kg) was injected to the mice intraperitoneally, and blood was collected from tail veins to measure glucose level at 15, 30, 60, 90, and 120 min. For IP-ITT, mice were administered intraperitoneal injections at a dosage of 1 U/kg. All other procedures were identical to those presented above. The trapezoidal method was used to calculate the area under the curve (AUC).

### 5.6. Indirect Calorimetry

Indirect calorimetry was performed using the Panlab Oxylet Pro System (Spain, Panlab LE 405). Animals were individually placed in metabolic cages, and respiratory measurements were recorded for three consecutive days at 20-min intervals following an adaptation period of 9 h. Then, the oxygen consumption rate (VO2; mL/kg/h), carbon dioxide production rate (VCO2; mL/kg/h), energy expenditure rate (EE; kcal/kg/h), respiratory exchange ratio (RER; VCO2/VO2), and activity were measured for 3 d. The energy expenditure rate was calculated using the following equation: Energy expenditure rate = 3.815 × VO2 + 1.232 × VCO2. The metabolic data were normalized regarding body weight.

### 5.7. Hematoxylin and Eosin (H&E) Staining

Freshly harvested adipose tissues were fixed in 4% paraformaldehyde, and an 8 μm cross-section of adipose tissue was processed with a standard procedure.

### 5.8. Quantitative Image Analysis

Representative images were acquired using a light microscope (Japan, Tokyo, Olympus, Olympus BX53). The average cell area and size of adipocytes were analyzed by Image J pro plus. Five fields were randomly selected in each sample, and the area and diameter of adipocytes were measured at 200× magnification. Average diameter was calculated from the number of measured adipocytes.

### 5.9. RNA Extraction, cDNA Synthesis, and Quantitative Real-Time PCR

Total RNA was extracted from frozen adipose tissues or cells using Trizol (Japan, TAKARA, #9109). cDNA was synthesized by reverse transcription of 1 μg RNA using the Prime Script First-Strand cDNA Synthesis Kit (Japan, TAKARA, #RR047A) according to the manufacturer’s instructions. The cDNA was quantified by qRT-PCR using the SYBR Green PCR Kit (Germany, Qiagen, #208054). The reaction was performed as follows: 95 °C for 5 min and 40 cycles of 95 °C for 30 s, 60 °C for 30 s, and 72 °C for 30 s. The reactions were conducted using an ABI QuantStudio TM 6 flex (United States, Applied Biosystems). Primers used in the PCR are listed in Supplementary Materials, Table S1. The comparative Ct (2^−ΔΔCt^) method was used. The Ct values were normalized to the β-actin gene or 18 s in the same sample.

### 5.10. Western Blotting

Total protein was extracted from adipose tissues and MEFs cells using RIPA lysis buffer with protease inhibitors. All samples were centrifuged at 12,000 r/min for 10 min at 4 °C, and the supernatants were collected. Protein concentrations were quantified by the Pierce^®^ BCA Protein Assay Kit (United States, Thermo Fisher, #23225). Proteins were separated with 10% SDS/PAGE gels and transferred to PVDF membranes. The membranes were probed with the following antibodies against PPARγ (China, ABclonal, #A0270), aP2 (China, ABclonal, #A0232), FAS (United States, CST, #3180), PGC-1α (China, ABclonal, #A11971), and β-actin (China, ABclonal, #AC004), then probed with HRP-conjugated secondary antibodies, and incubated with developing solution (United States, Bio-rad, #170-5060). β-actin was used as the internal reference.

### 5.11. Mouse Embryonic Fibroblasts (MEFs) and Adipogenesis

MEFs were isolated from E14.5 d embryos and maintained as previously described [49]. MEFs were treated with differentiation medium (10% fetal bovine serum (FBS) containing 0.5 μM 3-isobutyl-1-methylxanthine, 1 μM dexamethasone, 10 μg/mL of insulin, and 200 μM indomethacin) for 4–6 d (medium was replaced every 2 d), and then cultured in maintenance medium supplemented with 10% FBS and 10 μg/mL insulin for 2 d, followed by exchange with regular medium containing 10% FBS and incubated for four days (media were changed every 1 d). The cells had an adipocyte phenotype, and lipid droplets were visible; only cells with 90% or higher differentiation after 14 days were used.

### 5.12. Oil Red O Staining

Oil Red staining was performed according to the protocol [50]. Briefly, the fully differentiated MEF cells were washed twice with PBS and then fixed with 4% paraformaldehyde for 0.5–1 h. After removal of the paraformaldehyde, the cells were stained with oil red O dye (0.5 g of oil red O dry powder dissolved in 100 mL isopropanol then diluted with dd H_2_O in a 3:2 ratio) at 37 °C for 1 h. Followed by removal of the dye, washing was conducted twice in PBS. Finally, triglyceride accumulation was photographed with a Nikon microscope (Eclipse TS100; Nikon, Tokyo, Japan).

### 5.13. Statistical Analysis

In this study, all data were graphed as the mean ± SEM and were analyzed using GraphPad prism software. Student’s t test (unpaired 2-tailed) was employed for comparison between two groups. Two-way ANOVA was used to examine interactions between multiple variables. *p* < 0.05 is statistically significant and is denoted as * *p* < 0.05 or ** *p* < 0.01.

## Data Availability

No new data were created or analyzed in this study. Data sharing is not applicable to this article.

## Supplementary Materials

The following are available online at https://www.mdpi.com/article/10.3390/ijms22105390/s1. Figure S1. Zfp217^+/−^ mice exhibit no phenotypic alterations compared to Zfp217^+/+^ mice fed a normal chow diet (NC); Figure S2. Zfp217^+/−^ mice and Zfp217^+/+^ mice exhibit similar glucose tolerance and insulin sensitivity under NC; Figure S3. Zfp217^+/−^ mice exhibit no difference compared to the Zfp217^+/+^ mice in energy expenditure under NC; Figure S4. Detection of the five most likely off-target sites of sgRNA2’ in the genome based on PCR and agarose gel electrophoresis; Table S1 Quantification real-time PCR primer sequence; Table S2 Primer design for off-target site.
