# Supplementary material for: Partial Deficiency of Zfp217 Resists High-Fat Diet-Induced Obesity by Increasing Energy Metabolism in Mice
Source: Int J Mol Sci. 2021 May 20;22(10):5390. doi: 10.3390/ijms22105390 (PMC8161011; doi:10.3390/ijms22105390)
Supplement: Supplementary file 1 [file ijms-22-05390-s001.zip › ijms-1212653-supplementary.pdf]

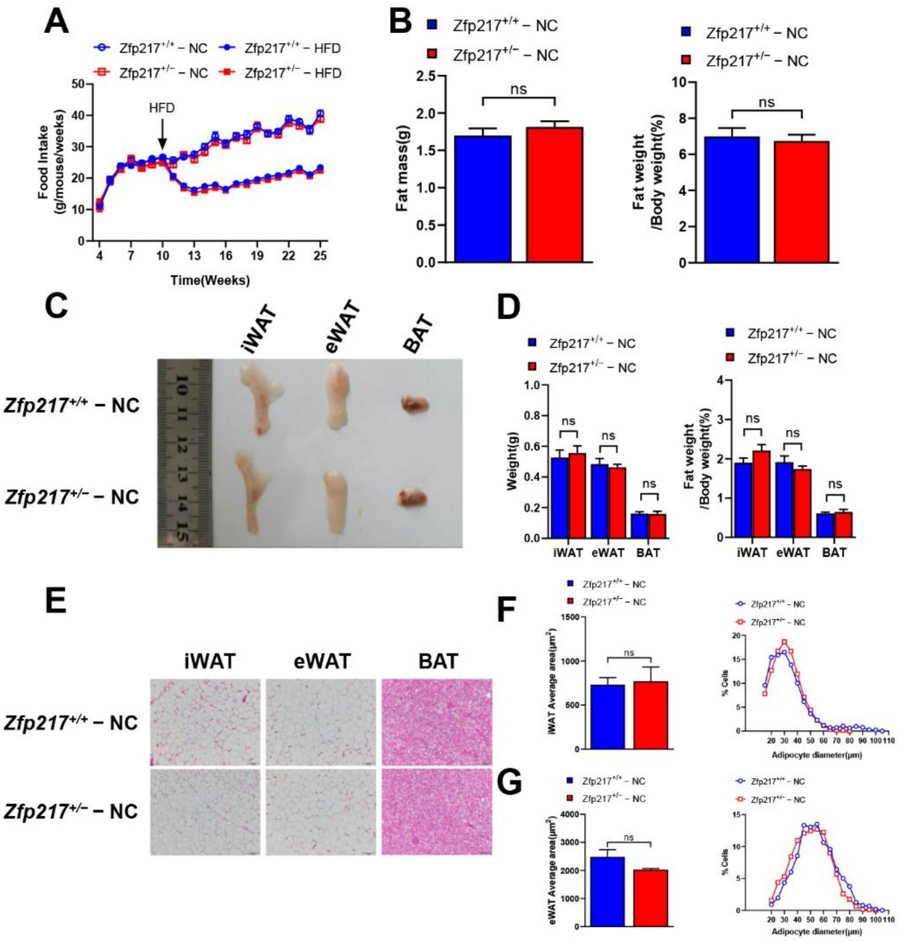

Figure S1. Zfp217$^{+/–}$ mice exhibit no phenotypic alterations compared to Zfp217$^{+/+}$ mice fed normal chow diet (NC). (A) Food intake of Zfp217$^{+/+}$ and Zfp217$^{+/–}$ mice under NC or HFD (n = 8 per group). (B) Body fat weight or percentage of Zfp217$^{+/+}$ and Zfp217$^{+/–}$ mice (n = 8 per group). (C) Gross appearance of adipose tissues from representative Zfp217$^{+/+}$ and Zfp217$^{+/–}$ mice at 26 weeks. (D) iWAT, eWAT, BAT weight or percentage of Zfp217$^{+/+}$ and Zfp217$^{+/–}$ mice (n = 8 per group). (E) H&E staining of adipose tissue from Zfp217$^{+/+}$ and Zfp217$^{+/–}$ mice (Scale bar: 100 μm) (n = 6 per group). (F, G) Average cell area (left) and adipocyte size distribution (right) in iWAT (F) and eWAT (G) of Zfp217$^{+/+}$ and Zfp217$^{+/–}$ mice (n = 6 per group). For all statistical plots, data are presented as the mean ± SEM. *P < 0.05; **P < 0.01 and ns indicates no significance between the two indicated groups.

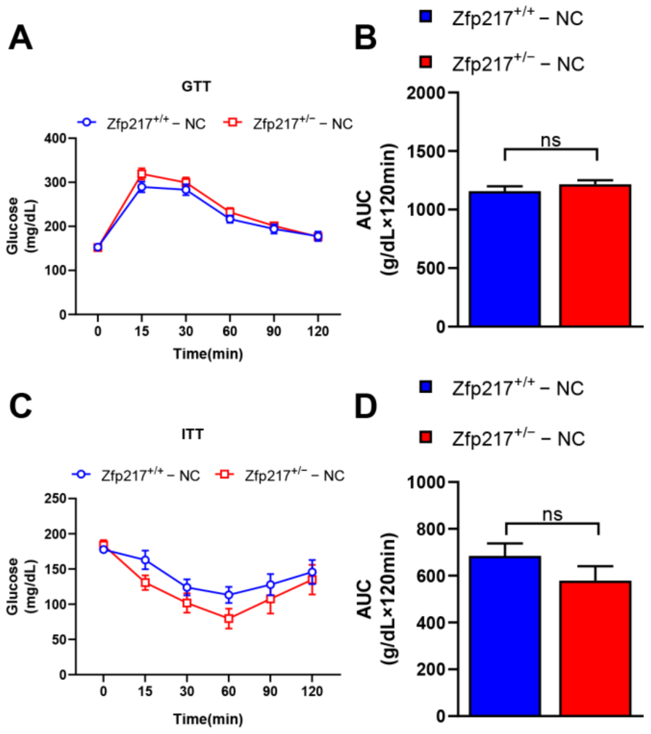

Figure S2. Zfp217$^{+/-}$ mice and Zfp217$^{+/+}$ mice exhibit similar glucose tolerance and insulin sensitivity under NC. (A, B) IP glucose tolerance test (GTT) were performed in Zfp217$^{+/+}$ and Zfp217$^{+/-}$ mice at 14 weeks, and the corresponding areas under the curves were calculated (n = 8 per group). Comparison at each time point was made against Zfp217$^{+/+}$ control mice by Two-way ANOVA. (C, D) IP insulin tolerance test (ITT) were performed in Zfp217$^{+/+}$ and Zfp217$^{+/-}$ mice at 15 weeks, and the corresponding areas under the curves were calculated (n = 8 per group). Comparison at each time point was made against Zfp217$^{+/+}$ control mice by Two-way ANOVA. For all statistical plots, data are presented as the mean ± SEM. *$P < 0.05$; **$P < 0.01$, and ns indicates no significance between the two indicated groups.

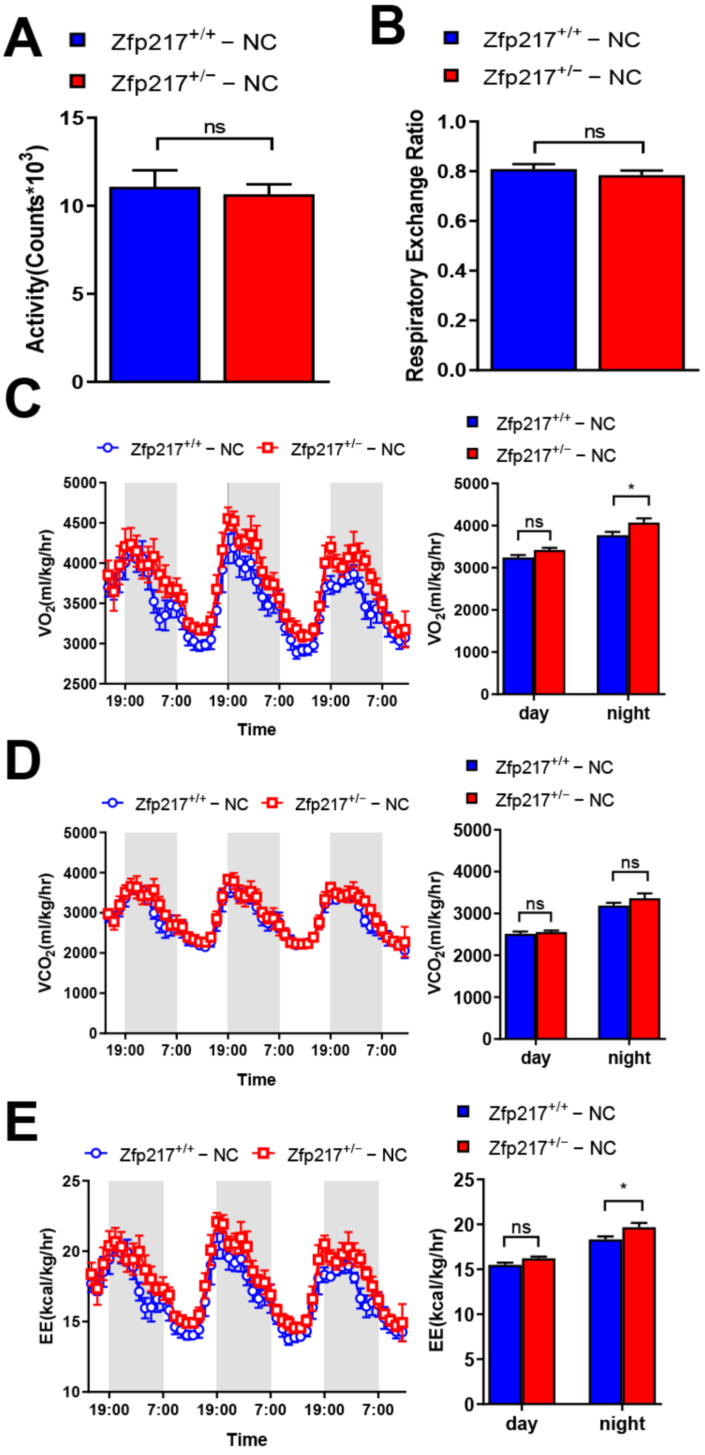

Figure S3. Zfp217$^{+/-}$ mice exhibit no difference compared to the Zfp217$^{+/+}$ mice in energy expenditure under NC. (A) Physical activity, (B) Respiratory exchange ratio, (C) Oxygen consumption (VO_2), (D) Carbon dioxide generation (VCO_2)

and (E) Energy expenditure analyzed by indirect calorimetry in Zfp217$^{+/+}$ and Zfp217$^{+/-}$ mice after NC feeding for 25 weeks (n=6 per group). For all statistical plots, data are presented as the mean ± SEM. *P < 0.05; **P < 0.01, and ns indicates no significance between the two indicated groups.

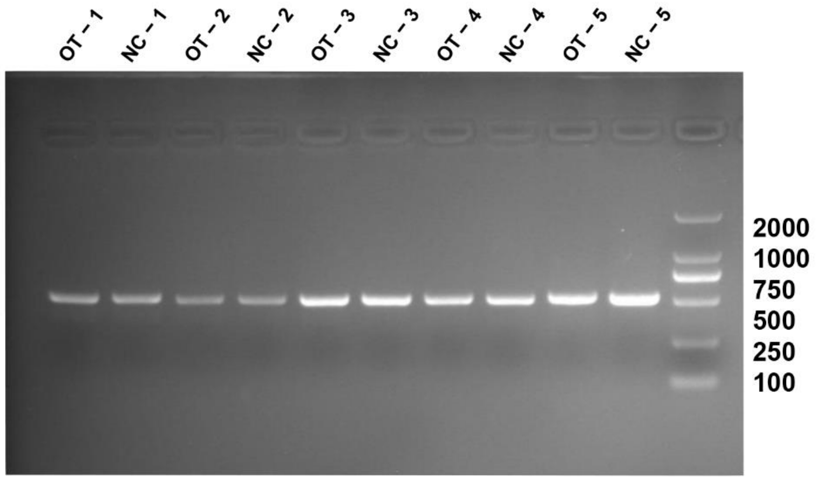

Figure S4. Detection of the five most likely off-target sites of sgRNA2' on the genome by PCR and agarose gel electrophoresis.

Table S1 Quantification real-time PCR primer sequence

Gene	Primer sequence	Retardation temperature,℃	Product length, bp
Zfp217	F: CTCTCTAGGCTCCCAGATGGA<br>R: AGAACATGCAATCCAGGGGC	60	131
PPARγ	F: TCGCTGATGCACTGCCTATG<br>R: GAGAGGTCCACAGAGCTGATT	60	103
Ap2	F: ATCAGCGTAAATGGGGATTTGG<br>R: GTCTGCGGTGATTTCATCGAA	60	105
C/EBPα	F: CAAGAACAGCAACGAGTACCG<br>R: GTCACTGGTCAACTCCAGCAC	60	124
adiponectin	F: TGTTCCTCTTAATCCTGCCCA<br>R: CCAACCTGCACAAGTTCCC	60	104
ACOX1	F: TAACTTCCTCACTCGAAGCCA<br>R: AGTTCCATGACCCATCTCTGTC	60	283
ACC1	F: ATGGGCGGAATGGTCTCTTTC<br>R: TGGGGACCTTGTCTTCATCAT	60	148
ELOVL6	F: GAAAAGCAGTTCAACGAGAACG<br>R: AGATGCCGACCACCAAAGATA	60	110
FAS	F: TATCAAGGAGGCCCATTTTGC<br>R: TGTTTCCACCTCTAAACCATGCT	60	195
Perlipin A	F: CTGTGTGCAATGCCTATGAGA<br>R: CTGGAGGGTATTGAAGAGCCG	60	171
HSL	F: GATTTACGCACGATGACACAGT<br>R: ACCTGCAAAGACATTAGACAGC	60	114
LPL	F: GGGAGTTTGGCTCCAGAGTTT<br>R: TGTGTCTTCAGGGGTCCTTAG	60	115
SCD1	F: TTCTTGCGATACACTCTGGTGC<br>R: CGGGATTGAATGTTCTTGTCGT	60	98

β-actin	F: CACGATGGAGGGGCCGGACTCATC<br>R: TAAAGACCTCTATGCCAACACAGT	60	241
18s	F: TTGACGGAAGGGCACCACCAG<br>R: GCACCACCACCCACGGAATCG	60	130

Table S2 Primer design for off-target site

Primer name	Primer sequence	Location on the genome
OT-Zfp217-1	F: AAGTTTAGGTAGCAGGAT<br>R: GATGGAAGAAATGGCAGA	Chr11: +93805835
OT-Zfp217-2	F: TGAGAAGGTTTTGGACTA<br>R: TTTCCCGTGTCACTTCAT	Chr18: -65234377
OT-Zfp217-3	F: GGACCTGGACCTGTAGTTT<br>R: CATGCCCAAGTATTACCC	Chr9: -44661009
OT-Zfp217-4	F: CTTCAGGCACATAGTCAGC<br>R: TACCTATTGGAGCCTTCT	Chr14: +9351328
OT-Zfp217-5	F: CCTTCCTCCCAGACCATC<br>R: AGGCCATTCTGGAACTCA	Chr16: -144443447
